# The Story of the Insane from Year to Year

**Published:** 1893-07-22

**Authors:** 


### THE STORY OF THE INSANE FROM YEAR TO YEAR.

(Fifth Series.)

### THE BARONY ASYLUM, LENZIE.

The sixteenth annual report of this asylum tells us it contained on January 1st, 1891, 559 patients, and on the last day of the year there were 568 in residence. The admissions were 194, the recoveries 65, and the deaths 59. These numbers show a percentage of 33 recoveries and of 10·36 death. Hereditary tendency and drink collectively account for 44 of the admissions, and 33 of the deaths were caused by brain diseases and nine by consumption. The asylum is now lighted by oil-gas and this system is said to be more economical and efficient than any other. English Medical Superintendents with an economical turn of mind would like to have some particulars of this new system. At the date of the report the asylum was overcrowded, but additions were being made to relieve this. "The Committee have to express their continued confidence in Dr. Blair," and Dr. Blair tenders his thanks to the Committee "for the kind support and encouragement received at their hands."

### BISHOPSTONE HOUSE, BEDFORD.

Some sixteen or seventeen years since, a Royal Commission sat on our lunacy system, and it was fully believed by some ill-informed people that many things would be disclosed adverse to asylums generally, and to private asylums in particular. These predictions were not destined to be fulfilled. Very little indeed was proved against any of them; against most of them nothing at all. Whether as a matter of principle private asylums should be allowed to exist is another question with which we are not now concerned, and as the last Lunacy Act placed the existing private asylums on a firmer basis than ever, we intend from time to time to devote some space in The Hospital for description of these "licensed houses," as they are legally called. The one named at the head of this notice is situated on the outskirts of the pleasant town of Bedford. It was licensed about fifteen years since for four patients. This number was soon increased to six, and subsequently to ten. The total number of patients received since its opening is 45. Of these, 18 have been discharged "recovered," six have been discharged "relieved," and four have died, three of the deaths being from senile decay. Eight have been transferred to other asylums from economical or other reasons, and nine remain in the asylum. The accommodation provided is very home-like in appearance, and the patients seem to have much freedom in many ways. Country walks or drives are of daily occurence, and it is only in the stages of excitement that the patients are limited to the grounds for exercise. The small number of patients received permits the closest supervision and the most careful individual treatment on the part of the superintendent and proprietor, Dr. Craig. Ladies only are received.

